# Secondary Use of COVID-19 Symptom Incidence Among Hospital Employees as an Example of Syndromic Surveillance of Hospital Admissions Within 7 Days

**DOI:** 10.1001/jamanetworkopen.2021.13782

**Published:** 2021-06-17

**Authors:** Steven Horng, Ashley O’Donoghue, Tenzin Dechen, Matthew Rabesa, Ayad Shammout, Lawrence Markson, Venkat Jegadeesan, Manu Tandon, Jennifer P. Stevens

**Affiliations:** 1 Center for Healthcare Delivery Science, Beth Israel Deaconess Medical Center, Boston, Massachusetts; 2 Department of Emergency Medicine, Beth Israel Deaconess Medical Center, Boston, Massachusetts; 3 Employee Health, Beth Israel Lahey Health, Boston, Massachusetts; 4 Information Systems, Beth Israel Deaconess Medical Center, Boston, Massachusetts; 5 Division for Pulmonary, Critical Care, and Sleep Medicine, Department of Medicine, Beth Israel Deaconess Medical Center, Boston, Massachusetts

## Abstract

**Importance:**

Alternative methods for hospital occupancy forecasting, essential information in hospital crisis planning, are necessary in a novel pandemic when traditional data sources such as disease testing are limited.

**Objective:**

To determine whether mandatory daily employee symptom attestation data can be used as syndromic surveillance to estimate COVID-19 hospitalizations in the communities where employees live.

**Design, Setting, and Participants:**

This cohort study was conducted from April 2, 2020, to November 4, 2020, at a large academic hospital network of 10 hospitals accounting for a total of 2384 beds and 136 000 discharges in New England. The participants included 6841 employees who worked on-site at hospital 1 and lived in the 10 hospitals’ service areas.

**Exposure:**

Daily employee self-reported symptoms were collected using an automated text messaging system from a single hospital.

**Main Outcomes and Measures:**

Mean absolute error (MAE) and weighted mean absolute percentage error (MAPE) of 7-day forecasts of daily COVID-19 hospital census at each hospital.

**Results:**

Among 6841 employees living within the 10 hospitals’ service areas, 5120 (74.8%) were female individuals and 3884 (56.8%) were White individuals; the mean (SD) age was 40.8 (13.6) years, and the mean (SD) time of service was 8.8 (10.4) years. The study model had a MAE of 6.9 patients with COVID-19 and a weighted MAPE of 1.5% for hospitalizations for the entire hospital network. The individual hospitals had an MAE that ranged from 0.9 to 4.5 patients (weighted MAPE ranged from 2.1% to 16.1%). For context, the mean network all-cause occupancy was 1286 during this period, so an error of 6.9 is only 0.5% of the network mean occupancy. Operationally, this level of error was negligible to the incident command center. At hospital 1, a doubling of the number of employees reporting symptoms (which corresponded to 4 additional employees reporting symptoms at the mean for hospital 1) was associated with a 5% increase in COVID-19 hospitalizations at hospital 1 in 7 days (regression coefficient, 0.05; 95% CI, 0.02-0.07; *P* < .001).

**Conclusions and Relevance:**

This cohort study found that a real-time employee health attestation tool used at a single hospital could be used to estimate subsequent hospitalizations in 7 days at hospitals throughout a larger hospital network in New England.

## Key Points

**Question:**

Can secondary use of employee symptom attestation data be used as syndromic surveillance to estimate COVID-19 hospitalizations in the communities where the employees live?

**Findings:**

In this cohort study of 6481 hospital employees, an increased frequency of COVID-19 symptoms reported by all employees at a single hospital was associated with increased hospitalizations across 10 hospitals 7 days later.

**Meaning:**

These findings suggest that in a novel pandemic before reliable testing is available, use of nontraditional secondary data sources can be used to estimate hospital demand.

## Teaser

This cohort study examines whether there is an association between data collected for suspected COVID-19 symptoms among hospital employees and COVID-19 hospitalizations within 7 days.

## Introduction

Given the limited testing for COVID-19 early in the pandemic, multiple businesses, including hospitals, required employees to report any symptoms associated with COVID-19 and directed symptomatic employees to obtain follow-up testing. Such symptom reporting tools may also have additional secondary benefits. The Department of Veterans Affairs described how their patients’ use of a symptom monitoring tool for COVID-19 improved a sense of connection,^1^ suggesting such employee attestation strategies may have additional benefits to health care organizations.

Prior efforts to have employees self-identify have been met with varied success.^2,3^ One critical weakness of symptom-only reporting is the infectivity of asymptomatic employees,^4^ who would only be identified with a larger surveillance testing strategy. As members of a community, however, hospital employees’ routine reporting of symptoms could serve as a surrogate of symptom reporting for their community as a whole. Given that prior research has noted that community spread of COVID-19 makes up the bulk of the burden of new infections in health care settings,^5^ employee attestations of symptoms may also have a secondary use as syndromic surveillance to estimate the incidence and prevalence of infections in the communities where employees live.

We hypothesized that an employee symptom reporting tool at a single academic medical center could be used as syndromic surveillance and forecast subsequent hospital admissions for COVID-19 in the communities where employees live.

## Methods

### Study Design

The Beth Israel Deaconess Medical Center institutional review board determined this cohort study to be exempt under category 4 because the identifiable health information was used for the purposes of health care operations and public health activities. A waiver of informed consent was granted as the research involves no more than minimal risk, could not practically be conducted without a waiver, and could not practically be conducted without personal health information. We followed the Strengthening the Reporting of Observational Studies in Epidemiology (STROBE) reporting guideline. Statistical analysis was performed in November 2020.

### Study Population

The study was performed in a large hospital system in Massachusetts, containing 10 hospitals from April 2, 2020, to November 4, 2020. The hospitals are numbered according to the number of unique employees living in that service area who are filling out the attestation during our sample period (ie, hospital 1 had the most employees living in their service area that fill out the employee attestation form whereas hospital 10 had the fewest). Hospital 1 is a tertiary, academic, teaching hospital in Boston, Massachusetts, with 719 staffed beds and 40 000 annual inpatient discharges. The entire network has 2384 staffed beds and 136 000 annual inpatient discharges.

### Inclusion and Exclusion Criteria

Employees were included if they were employed at the urban tertiary care hospital (hospital 1) and were working on-site that day. The self-reported symptoms were recorded by the institution when employees worked on-site for a given day. Employees were excluded if they lived outside of the 10-hospital network’s service area or if they were not working on-site that day. Attending physicians were members of a separate physicians’ organization and were excluded.

### Data Collection Tool

**Figure 1.**
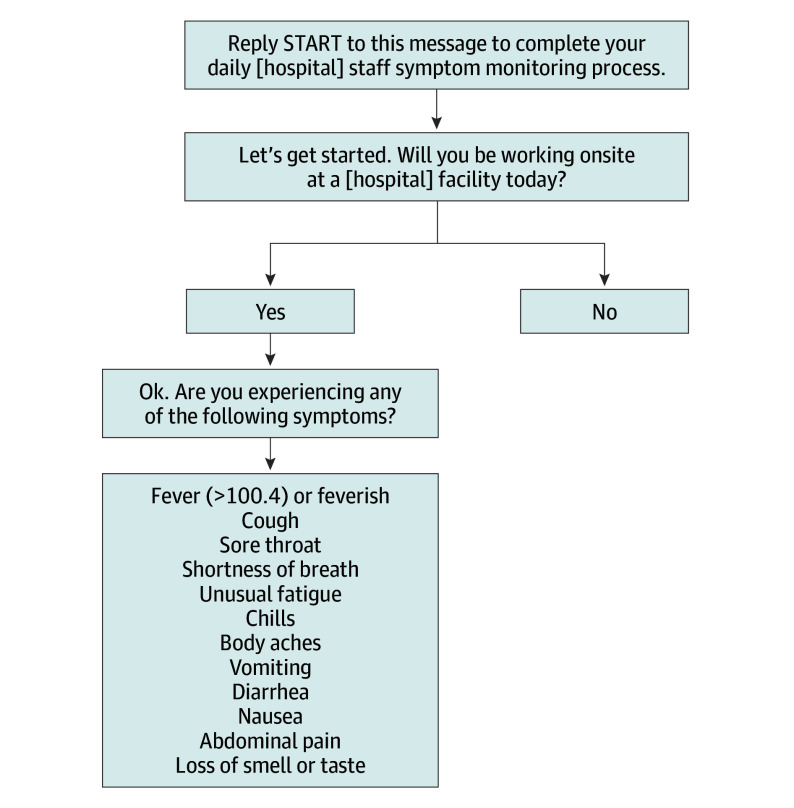
Employee Symptom Attestation Tool

Self-reported symptoms were collected using an automated text messaging system (Figure 1). Employees received a text message each morning, asking them to complete the daily symptom monitoring assessment. Although completion was not mandatory, it was strongly encouraged. Employees were first asked whether they will be working on-site that day. Only employees who reported that they would be working on-site were prompted to fill out the symptom reporting form, where they were asked if they were experiencing any COVID-related symptoms from a list of 12 symptoms. If yes, they were asked to report the specific symptom from the list of 12 that they were experiencing.

### Outcomes

Our primary outcome was the mean absolute error (MAE) and weighted mean absolute percentage error (MAPE) of 7-day forecasts of daily COVID-19 hospital census across all of the 10-hospital New England hospital network. We note that the primary outcome measure is not the mean of each individual hospital’s MAE, but rather the MAE of the network as a whole. Our secondary outcome was the MAE and weighted MAPE of 7-day forecasts of weekly COVID-19 positive cases within each of the 10 hospital service areas in a New England hospital network. This outcome has a reduced time period for analysis because of data availability from the state reporting agency. We also report the projected daily number of COVID-19 hospitalization patients for clarity.

### Study Variables

The role of the employee (registered nurse, operations, administrative support, research, clinical technician, housestaff/fellows/residents/interns, clinical assistants, and other), age, years of service, sex, race (White, Black, Asian, Hispanic, Other), and zip code were collected from employee records. The COVID-19 hospitalization data were sourced from the network’s incident command center, which aggregates and reports the COVID-19 hospitalization statistics to state and federal agencies following state and federal reporting guidelines.

### Data Processing

We collected the number of employees reporting any COVID-related symptom each day, grouped by employee home zip code. Employees’ home zip codes were then matched to the service areas of hospitals within the hospital network. Employees were therefore matched to the hospital nearest to which they live, rather than the hospital at which they work. The data were smoothed using a 7-day moving average (mean) due to differences in the numbers of employees filling out the attestation on weekends vs weekdays. To adjust for the changing variance in the data over time, it was transformed by taking the natural logarithm of the data (due to there being some days with observations of 0, we added 1 to the data as a monotonic transformation before taking the natural logarithm).

### Statistical Analysis

We report means and SDs of continuous variables and counts and percentages of categorical variables. We used an econometric time series forecasting model described by Dumitrescu and Hurlin^6^ to model each hospital as a cross-section of a panel, and test for Granger noncausality in this heterogeneous panel. This type of model has previously been used to estimate COVID-19 cases at the country level using Google search trends.^7^ In this framework, a linear autoregressive model is used, allowing coefficients to differ across hospitals in the panel, but fixed over time. Holding the number of lags constant across hospitals, we selected the optimal number of lags that minimizes the bayesian information criterion and estimated future COVID-19 hospitalizations at each network hospital. We found that the optimal number of lags is 7 days, which minimized the bayesian information criteria. We estimated a multivariable autoregressive linear regression model that included each hospital’s daily COVID hospital census, and the number of employees reporting symptoms in each hospital’s service area, with a lag of 7 days, to estimate the daily COVID hospital census for each hospital over 209 days. We estimated 7 days into the future and we calculated the MAE and weighted MAPE of our estimate to measure the accuracy of our model for the network. There were no missing days of hospital census data or employee symptom reporting data. As a secondary analysis, we used positive COVID-19 cases in a hospital’s service area, rather than hospitalizations, as the dependent variable.

*P* ≤ 0.05 was considered statistically significant and all tests were 2 tailed. Stata SE version 16 (StataCorp) was used for statistical analysis in November 2020.

## Results

Of 6841 employees included in the study, 5120 (74.8%) were female individuals, 3884 (56.8%) were White individuals, 1818 (26.6%) were registered nurses, 3085 (45.1%) lived in hospital 1’s service area, and 1147 (16.7%) reported a symptom at least once during the sample period; the mean (SD) age was 40.8 (13.6) years, and the mean (SD) time of service was 8.8 (10.4) years. Summary statistics about the employees are reported in Table 1.

**Table 1.** Descriptive Statistics of Employees

Characteristics	Employees, No. (%) (N = 6841)
Sex	
Female	5120 (74.8)
Male	1721 (25.2)
Age, y	
<20	13 (0.2)
20-29	1839 (26.9)
30-39	1799 (26.3)
40-49	1208 (17.7)
50-59	1157 (16.9)
≥60	825 (12.1)
Service time, y	
<10	4661 (68.1)
10-19	1164 (17.0)
20-29	523 (7.6)
30-39	383 (5.6)
≥40	110 (1.6)
Race	
White	3884 (56.8)
Black	1017 (14.9)
Asian	713 (10.4)
Hispanic	547 (8.0)
Other^a^	680 (9.9)
Roles	
Registered nurse	1818 (26.6)
Operations	1230 (18.0)
Administrative support	730 (10.7)
Research	597 (8.7)
Clinical technician	501 (7.3)
Housestaff/fellows/residents/interns	493 (7.2)
Assistant - clinical	476 (7.0)
Other^b^	996 (14.6)
Service area where employee lived^c^	
Hospital 1	3085 (45.1)
Hospital 2	1197 (17.5)
Hospital 3	733 (10.7)
Hospital 4	488 (7.1)
Hospital 5	457 (6.7)
Hospital 6	444 (6.5)
Hospital 7	270 (3.9)
Hospital 8	81 (1.2)
Hospital 9	64 (0.9)
Hospital 10	22 (0.3)

^a^ Other races included American Indian, Pacific Islander, 2 or more races, or other.

^b^ Other roles included clinical nurse specialist, case manager, dietician, imaging technologist, information technologist, nurse practitioner, nonclinical technician, physicians assistant, pharmacist, physicist, radiation therapist, rehab services (physical therapy/occupational therapy/speech and swallow), respiratory therapist, and social worker.

^c^ These were the hospital service areas where employees resided, not where they worked.

The total network had a mean (SD) COVID-19 census of 147.9 (168.7) patients and a mean (SD) of 11.2 (15.1) employees reporting symptoms each day. Hospital 1 had a mean (SD) COVID-19 census of 57.2 (61.5) patients and a mean (SD) of 4.8 (5.9) employees reporting symptoms each day, whereas hospital 10 had a mean (SD) COVID-19 census of 2.8 (3.5) patients and a mean (SD) of 0.1 (0.4) employees reporting symptoms each day. Employees filling attestations made up 0.8% of the total network’s weighted service area (where service area population is weighted by the hospital’s market share). Table 2 reports the descriptive statistics by hospital including the mean COVID-19 hospital census during the time of investigation and the mean number of employees reporting symptoms per day.

**Table 2.** Descriptive Statistics of Hospitals

Hospital	Staffed beds, No.	Annual inpatient discharges, No.	Daily COVID-19 hospitalizations, mean (SD)	Daily employees reporting symptoms, mean (SD)	Employees/service area population, %^a^	All-cause hospitalizations, mean (SD)	MAE (weighted MAPE)	MAE/all-cause hospitalization, %
Hospital 1	719	40 000	57.2 (61.5)	4.8 (5.9)	0.8	51.9 (3.8)	3.8 (2.7)	0.9
Hospital 2	208	13 000	10.3 (15.7)	1.9 (2.7)	4.6	11.0 (1.4)	1.4 (5.7)	1.4
Hospital 3	102	6000	8.1 (11.8)	1.3 (2.1)	1.6	9.5 (3.0)	3.0 (5.7)	4.8
Hospital 4	345	24 000	27.8 (36.4)	0.6 (1.3)	0.4	27.0 (3.7)	3.7 (4.3)	1.4
Hospital 5	164	11 000	13.0 (12.8)	0.6 (1.1)	0.5	14.1 (4.5)	4.5 (16.1)	3.8
Hospital 6	58	2500	4.0 (5.4)	0.6 (1.2)	2.0	5.2 (1.5)	1.5 (7.3)	4.5
Hospital 7	364	19 500	15.8 (17.5)	0.5 (1.0)	0.3	11.0 (1.3)	1.3 (3.6)	1.0
Hospital 8	205	12 000	8.6 (11.5)	0.1 (0.4)	0.3	14.8 (0.9)	0.9 (2.1)	1.0
Hospital 9	140	5500	0.0 (0.1)	0.1 (0.4)	2.7	8.1 (0.1)	0.1 (14.2)	0.2
Hospital 10	79	2500	2.8 (3.5)	0.1 (0.4)	0.2	3.9 (1.3)	1.3 (4.9)	7.3
Network	2384	136 000	147.9 (168.7)	11.2 (15.1)	0.8	137.8 (6.9)	6.9 (1.5)^b^	0.5

Abbreviations: MAE, mean absolute error; MAPE, mean absolute percentage error (mean absolute percentage error weighted by hospital census).

^a^ Number of employees living in hospital’s service area divided by the weighted service area’s population, (weighted by the hospital’s market share) displayed as a percentage.

^b^ Network MAE here describes the MAE of the network as a whole, not the mean of the individual hospital’s MAE.

**Figure 2.**
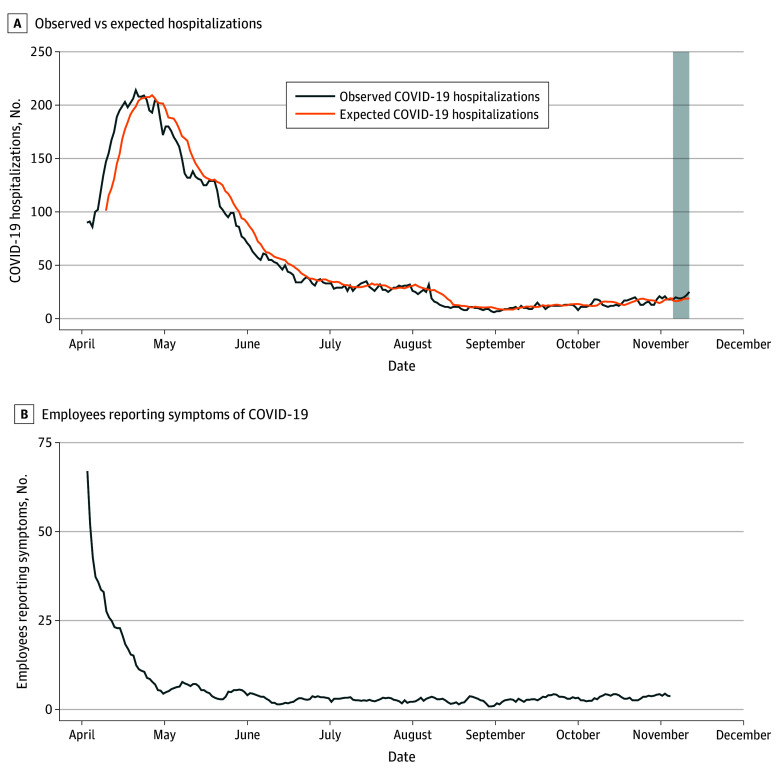
Expected Hospitalizations and Employee Symptom Reporting for Hospital 1 A, the shaded area denotes the 7-day period for which hospitalizations were estimated.

Figure 2A plots the observed COVID-19 hospital census at hospital 1 along with our expected values over a 7-day period. COVID-19 hospitalizations began at 90 patients and reached a peak of 214 patients on April 21, 2020; this number then decreased to 18 patients by November 4, 2020 (Figure 2A). Figure 2B plots the number of employees reporting symptoms who lived in the service area of hospital 1 each day. It began with 67 employees reporting symptoms and quickly decreased to 4 employees reporting symptoms by May 1, 2020. From May 2020 onwards, there was a mean (SD) of 3.30 (2.55) employees reporting symptoms.

Using a Granger noncausality test, we rejected the null hypothesis that the number of employees reporting symptoms was not useful for estimating future COVID-19 hospitalizations at any of the in-network hospitals (*Z̃* statistic = 4.63; *P* < .001). We found that the optimal number of lags was 7 days, which minimized the bayesian information criterion.

Among larger hospitals and those with greater numbers of employees in their service areas, the employee symptoms were associated with increases in hospitalizations due to COVID-19. The regression coefficient on hospital 1’s 7-day lag was 0.05 (95% CI, 0.02-0.07; *P* < .001), which can be interpreted as meaning that twice as many employees reporting symptoms today was associated with a 5% increase in COVID-19 hospitalizations in 7 days. At the mean for hospital 1, this would correspond to 4.8 additional employees reporting symptoms today and 3 additional COVID-19 hospitalizations in 7 days. For hospital 2, 7-day lag of symptoms was not statistically significantly different from 0. For hospital 3, a doubling of employees reporting symptoms today (1.3 additional reports at hospital 3′s mean) was associated with a 6% increase in COVID-19 hospitalizations in 7 days (regression coefficient, 0.06; 95% CI, 0.00-0.12; *P* < .001). For hospital 4, a doubling of employees reporting symptoms (0.6 additional reports at hospital 4’s mean) was associated with an 8% increase in COVID-19 hospitalizations in 7 days (regression coefficient, 0.08; 95% CI, 0.01-0.16; *P* < .001). For hospital 5, a doubling of employees reporting symptoms (0.6 additional reports at hospital 5’s mean) was associated with an 8% increase in COVID-19 hospitalizations in 7 days (regression coefficient, 0.08; 95% CI, 0.02-0.14; *P* < .001). For hospitals 6 through 10, the 7-day lag of symptoms was not statistically significant. In Table 2, we report the MAE and weighted MAPE by hospital for estimates from November 5 to November 11, 2020. The network model had a MAE of 6.9 patients for the network. This corresponded to a weighted MAPE of 1.5%. The individual hospitals had an MAE that ranged from 0.9 to 4.5 patients (weighted MAPE ranged from 2.1% to 16.1%). Hospital 1’s MAE was 3.8 patients (weighted MAPE = 2.7%). For context, the mean network all-cause occupancy was 1286 during this period, so an error of 6.9 is only 0.5% of the network mean occupancy. As a secondary analysis, we also tried to estimate positive cases, not just hospitalizations. We did this because the lag time between symptom onset and COVID-19 test positivity is shorter than symptoms onset and hospitalization. However, this method relies more heavily on adequate and accessible testing, which is why hospitalizations are our preferred outcome measure of interest. Furthermore, the state reporting agency only reports weekly case numbers by town, and only began reporting those numbers at the end of April. Thus, this analysis is done on a reduced sample at the week level. From a Granger noncausality test for panel data, we cannot reject the null hypothesis that employees reporting symptoms is not useful for estimating changes in positive cases in the employees’ home community (*Z̃* statistic = 1.20; *P* = .15)

## Discussion

With inconsistent access to broader testing that has varied over the course of the COVID-19 pandemic, many hospitals have relied on employees to accurately identify themselves as having symptoms of COVID-19 infections. In this study, we found a secondary use of these data to estimate future hospitalizations in 7 days in the communities in which these employees lived.

There is considerable prior work in epidemiological modeling as well as forecasting to better understand, estimate, and anticipate health care delivery needs during the COVID-19 pandemic. A common approach used was a more traditional epidemiological-derived model, the Susceptible-Exposed-Infected-Recovered (SEIR). In June 2020, several prominent SEIR national models averaged a mean absolute percentage error of 32.6%.^8^ Alternatively, another common COVID-19 forecasting approach was to use data from earlier outbreaks, adapting another locations curve to the local area, as the University of Washington’s Institute for Health Metrics and Evaluation model had done, yielding the lowest MAPE during this time period at 20.2%.^8^ However, these are national models that estimate further (10 weeks) into the future and estimate deaths due to COVID-19. The dynamic, rapidly changing COVID-19 policies for social distancing are challenging for these alternative approaches as they are highly sensitive to any change in transmissibility. In both of these alternative approaches, modelers must estimate not only the effect of any of these policy changes on the transmissibility of COVID-19, but also the rigor of their enforcement and adherence by the community. We sought a different approach, which required no knowledge or estimation of ongoing policies, was highly localized to a specific area, was designed to estimate only into the immediate future (1 week), and forecasted hospitalizations, not deaths. Although our method restricted our estimation capability to the near future (7 days), which may not be sufficient for state-level policy decision making, a 7-day window is sufficient to activate initial hospital and network emergency surge protocols. For example, New York state has mandated hospitals have a surge and flex response plan to expand operational bed capacity by 50% within 7 days.^9^

In future work, we plan to investigate other nontraditional data sources used previously in syndromic surveillance that may also act as surrogates for community spread. For example, when one is symptomatic with other types of infectious diseases such as influenza, one may make purchases of items to manage those symptoms such as facial tissues, orange juice, and other over-the-counter products.^10^ It is plausible that other over-the-counter remedies that may be associated with COVID-19, such as acetaminophen, aspirin, and vitamin D, could also be used. We also plan to investigate alternative methodological approaches. For example, we currently allow hospitals to have individual coefficients, independent of other hospitals. The coefficients are unlikely to be exactly the same, because hospitalization rates are likely to differ across communities, reflecting the diversity of demographics and underlying risk factors in different communities. However, these coefficients are unlikely to be entirely independent, so some soft parameter sharing across hospitals may be warranted.

### Limitations

Our study does have several notable limitations. First, our estimate of COVID-19 daily hospital census represents the number of confirmed cases at each hospital. Thus, this estimate relies on testing data. Second, our independent variable relies on self-reported symptoms of employees reporting on-site for work. This depends on employees diligently filling out the symptom reporting and being honest about their experienced symptoms. Although every effort is made to ensure all employees reporting to work are submitting an employee attestation form before they arrive, some employees may be on-site without filling out the employee attestation form. Employees may inconsistently report symptoms based on day of week, access to sick leave, and individual level of concern, all which create different patterns of missingness and bias in the attestation data itself. Although our hospital makes a conscious effort to recruit employees from the community we serve, our data would only be representative of the working population and less representative of the nursing home population, inmates at prisons, and other members of the community unlikely to be working at a hospital. However, our findings suggest that despite these limitations and biases, employee-reported symptoms still provide an adequate signal.

Lastly, although we implemented employee attestations at all of our hospitals, we were only permitted access to those records for hospital 1. We reported the MAE for each individual hospital area, but the number of employees reporting from the other hospital service areas was low, so we focused our results and discussion only at the network level and for hospital 1, which closely mirrors how these forecasts were used by our incident command center.

## Conclusions

This cohort study found an example of syndromic surveillance, a tool of particular utility during pandemic conditions where decision making and health care resources need to be mobilized rapidly with imperfect and inconsistent information. Although employee attestation data are not perfect, and in fact are both biased and not completely representative of the community as a whole, our results suggest that such an approach was useful for the purposes of actionable operational decisions in surge planning during a pandemic, specifically when more accurate data were unavailable.
